# Latest development in the fabrication and use of lignin-derived humic acid

**DOI:** 10.1186/s13068-023-02278-3

**Published:** 2023-03-07

**Authors:** Shrikanta Sutradhar, Pedram Fatehi

**Affiliations:** Biorefining Research Institute, Lakehead University, 955 Oliver Road, Thunder Bay, ON P7B 5E1 Canada

**Keywords:** Lignin, Humic acid, Fertilizer, Humification, Oxidation

## Abstract

Humic substances (HS) are originated from naturally decaying biomass. The main products of HS are humic acids, fulvic acids, and humins. HS are extracted from natural origins (e.g., coals, lignite, forest, and river sediments). However, the production of HS from these resources is not environmentally friendly, potentially impacting ecological systems. Earlier theories claimed that the HS might be transformed from lignin by enzymatic or aerobic oxidation. On the other hand, lignin is a by-product of pulp and paper production processes and is available commercially. However, it is still under-utilized. To address the challenges of producing environmentally friendly HS and accommodating lignin in valorized processes, the production of lignin-derived HS has attracted attention. Currently, several chemical modification pathways can be followed to convert lignin into HS-like materials, such as alkaline aerobic oxidation, alkaline oxidative digestion, and oxidative ammonolysis of lignin. This review paper discusses the fundamental aspects of lignin transformation to HS comprehensively. The applications of natural HS and lignin-derived HS in various fields, such as soil enrichment, fertilizers, wastewater treatment, water decontamination, and medicines, were comprehensively discussed. Furthermore, the current challenges associated with the production and use of HS from lignin were described.

## Introduction

Although the global population has been increasing at an alarming rate, agricultural land has not expanded significantly [1, 2]. In this circumstance, improving the human ability to grow grains in a limited space, e.g., in small fields, is critical. Farmers depend on inorganic chemical fertilizers to keep the soil fertile for cultivation. However, the overused lands become unfertile and saline with a different pH in the long run. Soil salinity is characterized by high amounts of Na^+^, Mg^+2^, Ca^+2^, Cl^–^, HCO_3_^–^, and SO_4_^–2^, affecting plant growth [3]. Moreover, the total carbon content in the soil decreases daily. The organic matter of soil contains the residues of plants and animals and other organic compounds that form during the biomass decomposition processes in the soil. In this case, about 60% of the organic matter of soil is humic substances (HS) [4–7], which play a vital role in the health of soil for cultivation.

HS are mainly composed of humic acids (HAs), fulvic acids (FAs), and humins [8]. Structurally, although HA and FA share similar functional groups, FA has a lower molecular weight than HA does. As HS are the oxidized products of degraded biomass (e.g., lignin), they contain many oxygen-containing functional groups, such as aliphatic/phenolic hydroxyl groups, carboxylic acid groups, and quinones [9, 10]. These materials can probably be fabricated from other materials.

HS can play a vital role in managing the actual organic content of the soil. However, their complicated chemical structures are not easily degraded by the soil's microorganisms. Moreover, their close interaction with soil minerals helps them remain intact for an extended period. Organic fertilizers, such as composts and cattle manures, are primarily used to balance the humus and mineral content and act as natural pesticides [11]. Like organic fertilizers, humic substances (HS) are used in a few countries to improve soil quality [3]. It is well documented that the HS play a vital role in atmospheric nitrogen management by increasing the soil's exchangeable NH_4_^+^ and available NO_3_^−^, thus preventing nitrogen leaching and stimulating nitrifying bacteria [12–14]. Moreover, complexation reactions by HS hinders the precipitation of soil minerals, such as iron and aluminum [15–18]. Previous studies also claimed that HS could form complexes with soil minerals (including toxic metals), hydroxides, and organic compounds [19–22]. However, the sources of natural HS are limited. Thus, the incentives for generating HS artificially from natural biopolymers, such as lignin, are high.

Lignin is the most abundant aromatic biopolymer on earth, containing many active functional groups, e.g., aliphatic and phenolic moieties. Lignin is a three-dimensional, highly cross-linked macromolecule composed of three substituted phenols of coniferyl, sinapyl, and p-coumaryl alcohols generated by enzymatic polymerization, yielding a vast number of functional groups and linkages [23, 24]. The primary source of lignin is plant biomass [24–27], mainly produced as the by-product of the pulping processes of wood and other plant resources. The chemical characteristics of lignin differ depending on the pulping processes and the origin of the lignin resources. Although unmodified lignin has a limited application today, many applications have been proposed for chemically modified lignin derivatives, such as fine chemicals, emulsifiers, flocculants, synthetic floorings, sequestering, binders, thermosets, paints, adhesives, and fuels [28–34]. There are various ways to modify lignin for valorization, such as pyrolysis [35–37], hydrolysis [38, 39], hydrogenolysis [40–42], gasification [43, 44], hydrothermal conversion [45], and oxidation [46–48]. Oxidation is the most popular route for lignin modification and depolymerization for vanillin and organic acid production [49, 50]. Oxidation can be conducted using different oxidizing agents or various catalysts and enzymes [47–49, 51–53]. Alkaline aerobic oxidation could be an efficient chemical process to convert lignin and lignocellulosic biomass into HS.

Earlier studies reported a direct connection between natural humification and lignin due to aromatic structures and other common functional groups found in HS and lignin [54, 55]. It was also illustrated that artificial humification by alkaline oxidation or oxidative ammonolysis/ammoxidation of technical lignin would be possible [56–61]. This review article describes the complete historical origin of HS and the similarities between HS and lignin comprehensively. Also, the natural humification process and recent approaches to transforming lignin into HS-like materials are extensively discussed. Furthermore, this review article extends the discussion on the application of lignin-derived HS.

### Origin of humic substances: historical review

Humic substances were first defined in 1761 by Wallerius as a decomposed organic matter [62]. In 1786, Achard extracted a brown substance from soil and peat using a KOH solution and named it humic acid [63, 64]. Humus, a Latin word suggesting a soil-like substance, was first introduced by de Saussure in 1804, referring to dark soil organic matter [62]. In 1837, Sprengel developed several methods for preparing humic acid by pretreating soil with dilute mineral acids before alkaline extraction [62]. Sven Oden (1919) postulated that HS are the light to dark-brown substances of unknown materials, which are formed in nature by the decomposition of organic matter through the actions of microorganisms or in a laboratory by oxidizing chemical reagents. Alternatively, it was suggested that humus is the product of the condensation reaction between carbohydrates and amino acids in a microorganism-free environment [65]. It was also stated that phenol, quinone, and hydroquinone oxidation in an alkaline solution yields compounds similar to humic acids [66].

**Fig. 1:**
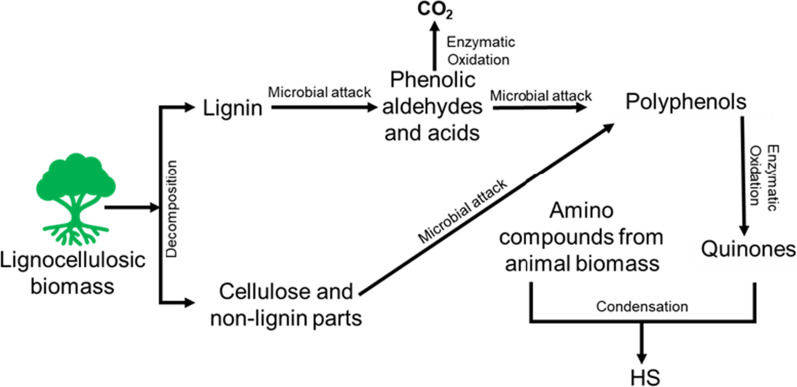
Polyphenol theory of HS formation from biomass adapted and redrawn from [67]

In 1936, Waksman proposed the “Lignin-protein theory” and stated that HS could be generated from the microbial attack of lignin [64]. According to this theory, the incomplete microbial attack of lignin molecules fragments lignin into smaller units and residues, which become part of the soil humus. In the degradation process, the methoxyl groups of lignin decompose into o-hydroxy phenols, and the oxidation of the aliphatic side chain converts into carboxylic acid groups. Moreover, Waksman reported that the presence of nitrogen compounds in the HS might result from the condensation of lignin with the microbial protein and other nitrogenous compounds. However, the final transformation of modified lignin residues to humic acids followed by fulvic acids was unclear in theory. Although the concept of Waksman's theory is controversial to many researchers, scientists agree with the theory that HS originate from plant residues and lignin-based materials. In 1982, Stevenson proposed the polyphenol theory of HS generation, as presented in Fig. 1. According to this theory, lignocellulosic biomass decomposes into lignin, cellulose and other non-lignin compounds (tannins, flavonoids, carotenoids, etc.). The lignin is fragmented into phenolic aldehydes and acids by the action of soil microorganisms. Some parts of these phenolic compounds (mainly phenolic acids) may oxidize to carbon dioxide by different enzymes. Later, these phenolic and non-lignin compounds are attacked by soil microorganisms and transformed into polyphenols. By enzymatic oxidation, the polyphenols convert to quinones. Finally, condensation occurs between animal protein amino compounds/acids in the soil and the quinones to transform into the natural HS in the soil [55].

**Fig. 2:**
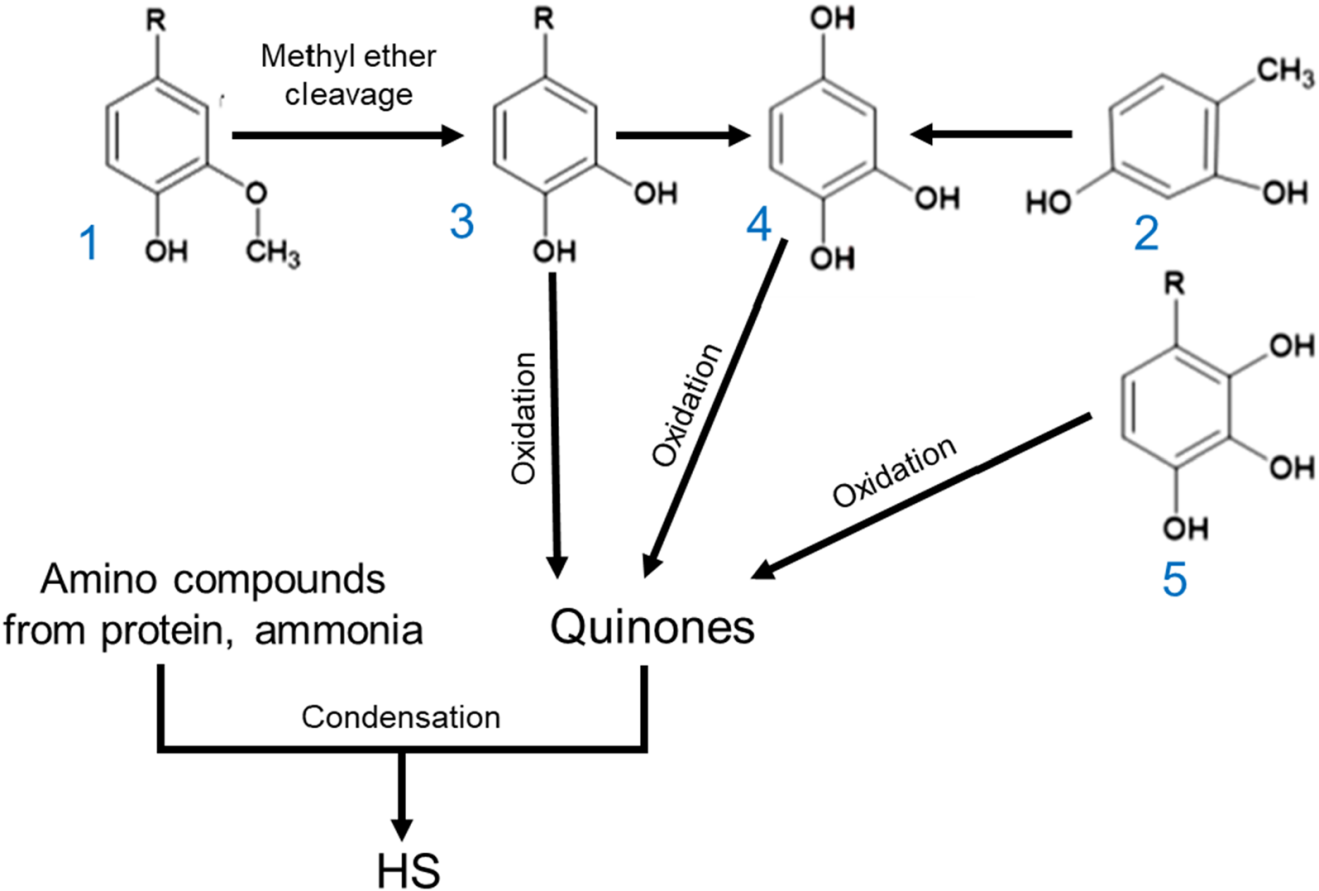
Reaction scheme for natural humification adapted and redrawn from [68]

In 1988, Flaig proposed a model reaction scheme for a natural humification process (Fig. 2). According to the model, the lignin macromolecule would fragment into precursors (1). Through microbial action and demethylation, the lignin units and other phenolic compounds from non-lignin parts (2, 3) would convert to catechols (4, 5). Further aerobic or enzymatic oxidation of those compounds would lead to quinone formation. Following condensation reactions, the amino acids from proteins and ammonia (degraded from protein by anaerobic digestion) would react with the quinones to transform into dark-brown HS polymers containing nitrogen [68]. It is also postulated that lignin's carbon and methoxyl contents would degrade, and other functional groups, such as hydroxyl, carbonyl, and carboxylic acid, would increase due to oxidation reactions. It was reported that when oxidized under pressure, lignin is converted to humic acid-like compounds and finally to aromatic compounds containing acid groups [50, 59].

### Properties of HS

**Fig. 3:**
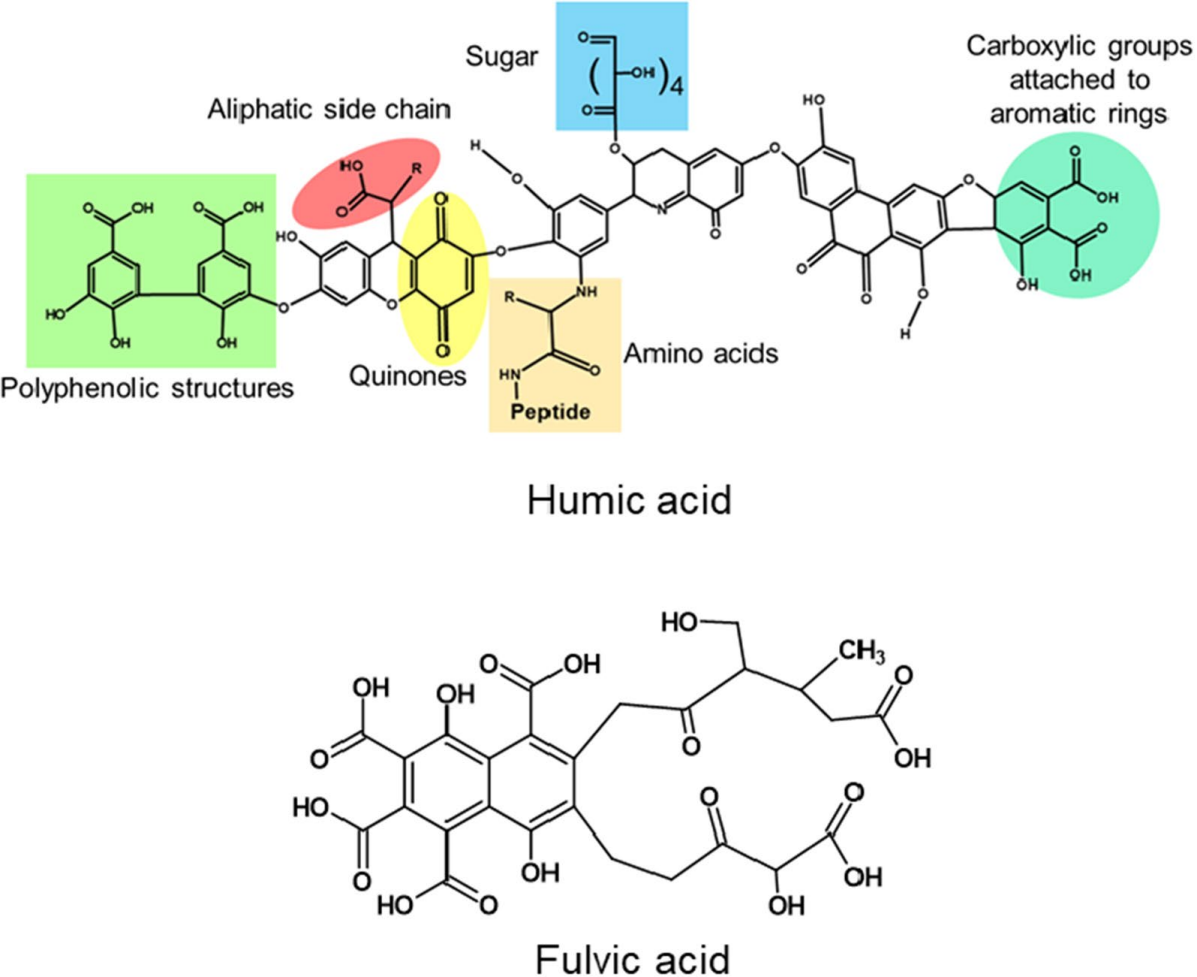
Chemical structures of Humic acid (HA) and fulvic acid (FA); adapted and redrawn from [67]

The origin, location, and extraction methods are the main factors that are responsible for the different chemical properties of HS [69]. The main constituents of HS are humin, HA, and FA. Figure 3 represents the tentative structures of HA and FA, while Table 1 describes the physicochemical properties of these compounds.

**Table 1:** Chemical properties of humin, HA, FA, and different types of lignin

Type of lignin	Solubility	Carboxylic acid group, mmol/g	Phenolic OH, mmol/g	Aliphatic OH, mmol/g	Molecular weight (Mw, g/mol)	C/N	Refs.
Humin	Not soluble	3–4	2	NA	> 300,000	NA	[69, 73–75]
HA	pH > 2	2–5	2–6	1–4	2000–1,000,000	8–61	[69, 73–76]
FA	soluble	8–9	3–6	3–5	600–900	6.7–9.2	[69, 73–75]
Kraft lignin (KL)	pH > 7	0.3	2.6	2.45	1000–15,000	135	[51, 77, 78]
Lignosulfonate (LS)	soluble	0.1–0.53	1.5–2	1.9–4	1000–50,000	240	[51, 78–80]
Organosolv lignin	pH > 7	0.05–0.25	2.6–5.1	1.3	500–5000	203	[53, 78, 79, 81]
Soda lignin	pH > 7	0.9–1	2.5–3.7	2.4	800–3000	68	[53, 78, 79, 82]

Humins are the insoluble fractions of HS, whereas HA and FA are the soluble fractions. The solubility of HA is pH dependent (Table 1). When the HA is dispersed in alkaline solutions, deprotonation happens, and the anionic hydrophilic groups, such as carboxylate and phenolates, dissociate in the solutions. On the other hand, in acidic media, due to protonation, HA precipitates [69, 70]. FA has a smaller degree of polymerization, less organic carbon, more oxygen contents, and high acidity; consequently, their solubility is higher compared to HA [71].

Depending on the source, HA has a wide range of functional groups, such as carboxylic, hydroxylic (both aliphatic and aromatic), quinones, amino acid groups, and carbohydrates [72]. Due to the significant amounts of carboxylic and phenolic OH, HA and FA show acidic behavior (Table 1), and HA has comparatively higher molecular weights than FA (Table 1).

The carbon-to-nitrogen ratio (C/N) is one of the essential properties of HS. Due to microbial action, degradation, and condensation with the amino compounds in the soil, natural HS are enriched with nitrogen. Therefore, the nitrogen content is higher in HA and FA than that in lignin (Table 1). Also, a smaller C/N is better for plant habitat applications, including agricultural land.

### Humification of waste biomass and non-lignin biomass materials

Table 2 describes recent developments in the transformation of waste biomass and non-lignin biomass materials into HS by hydrothermal (HT) and alkali pre-treatment [83–90]. A two-stage HT process (200 ℃) was developed and successfully generated 28 wt.% of HA from corn stalks [83]. The study reported that transforming biomass to HA by HT depends on the pH of the solution. In the first stage of acidic HT, the corn stalks generated precursors, such as carbohydrates, furans, phenols, and different organic acids. Later, the alkaline HT process converted these precursors into artificial HA. An earlier study also reported that, under acidic conditions (pH 1 to 5), the carbohydrates (i.e., glucose or saccharides) would be converted to 5-hydroxymethyl-furfural-1-aldehyde (HMF) through dehydration [84]. A condensation reaction would combine organic acids with the HMF to generate branched HS-like products (HA and FA) [84].

**Table 2:** Humification of biomass and non-lignin materials by alternative methods

Raw material	Chemical processes	Conditions	Yield	Refs.
Corn stalk	Two-stage Hydrothermal	180 ℃, 4 h, pH 1 180 ℃, 4 h, pH 13	HA-28.7%	[83]
Wheat straw	Hydrothermal	220 ℃, 4 h	HA-30.2%	[88]
Broccoli stem	Hydrothermal	204–220 ℃, 10 min	HS-198 g/kg HA-50.7 g/kg FA-28 g/kg	[89]
Sugarcane exocarp	Hydrothermal	200 ℃, 1 h	HA-14.85%	[86]
Cabbage leaf	Alkali- Hydrothermal	KOH (25%), NH4OH (20%), 195 ℃, 4 h	Not available	[87]
Glucose, saw dust, tulip tree leaves	Alkali-Hydrothermal	KOH	HA-1.8%	[84]
Food wastes (rice, meat, cabbage, potatoes)	Hydrothermal	215 ℃, 1 h	HA-43.5	[91]
Fermented Furfural	Alkali dissolution and acidification	KOH (8%) 70 ℃, 2.5 h	HA-49%	[85]
Carbohydrates monomer	Hydrothermal	([BMIM]Cl) (10 g) CrCl_3_ (0.74 g) 110 ℃, 4 h	HA-56.6%	[92]

The reaction conditions affect the characteristics of HA production greatly. Generally, insoluble HS (humins) formations would be dominant under acidic conditions, while soluble HA would be formed under alkaline conditions [90]. The yield of HS (HA or FA) in HT processes also depend on the reaction temperature. An earlier study reported that increasing temperature increased the HS formation. In this context, increasing the temperature from 184 to 220 ℃ in the HT treatment of broccoli stem resulted in HA yield elevation from 30.9 to 50.7 g/kg [89]. Moreover, alkaline HT processes toward the formation of HA depend on the strength of the alkali. The effect of different alkalis, such as KOH and NH_4_OH, was studied to observe the HA formation from cabbage leaves and reported that a strong alkali increased the HA yield due to the higher delignification rate [87]. The main drawback of the direct alkali HT process (Table 4) is the lower yield (1.8–2.3%), which might hinder the formation of HMF in a high alkaline environment. Few studies reported the neutral HT treatment (water) of waste biomass (i.e., wheat straw, sugarcane exocarp and food wastes) and reported a significant yield of HA (15–44%) [86, 88, 91]. Due to the self-ionization at a high temperature, water can generate H^+^ ions that hydrolyze the macromolecules (i.e., cellulose, hemicellulose, lignin, and protein) of biomass to their monomers (i.e., glucose, xylose, HMF, phenolic monomers, formic acid, lactic acids, amino acids, etc.) [86, 91]. Furthermore, under the acidic environment (generated organic acids), amino acids, phenolic compounds, and the HMF derivatives may polymerize to form HS [91]. The HT process is carried out at a high operating temperature (Table 2) to generate the HMF, which is considered one of the essential precursors for HS formation.

In addition to those acidic and alkaline HT, carbohydrates monomers (i.e., glucose, fructose) can be converted to HS through HMF formation in the presence of different ionic liquids, such as 1-butyl-3-methylimidazolium chloride ([BMIM]Cl), with transitional metal salts as catalysts (i.e., CrCl_3_) [92] at a comparatively lower temperature. Xu et al. reported the production of water-soluble humins (HA) could be achieved by 56.6% at 110 ℃ [92]. The alkali treatment (8% KOH solution) of pre-fermented furfural (FR) residue could also be utilized for artificial humification, which would be followed by acidification to achieve a material with 49% HA [85]. However, the formation of humins from carbohydrates would be considered an undesirable by-product that reduces the yield of HMF [92].

### Lignin: types, properties, and applications

**Fig. 4:**
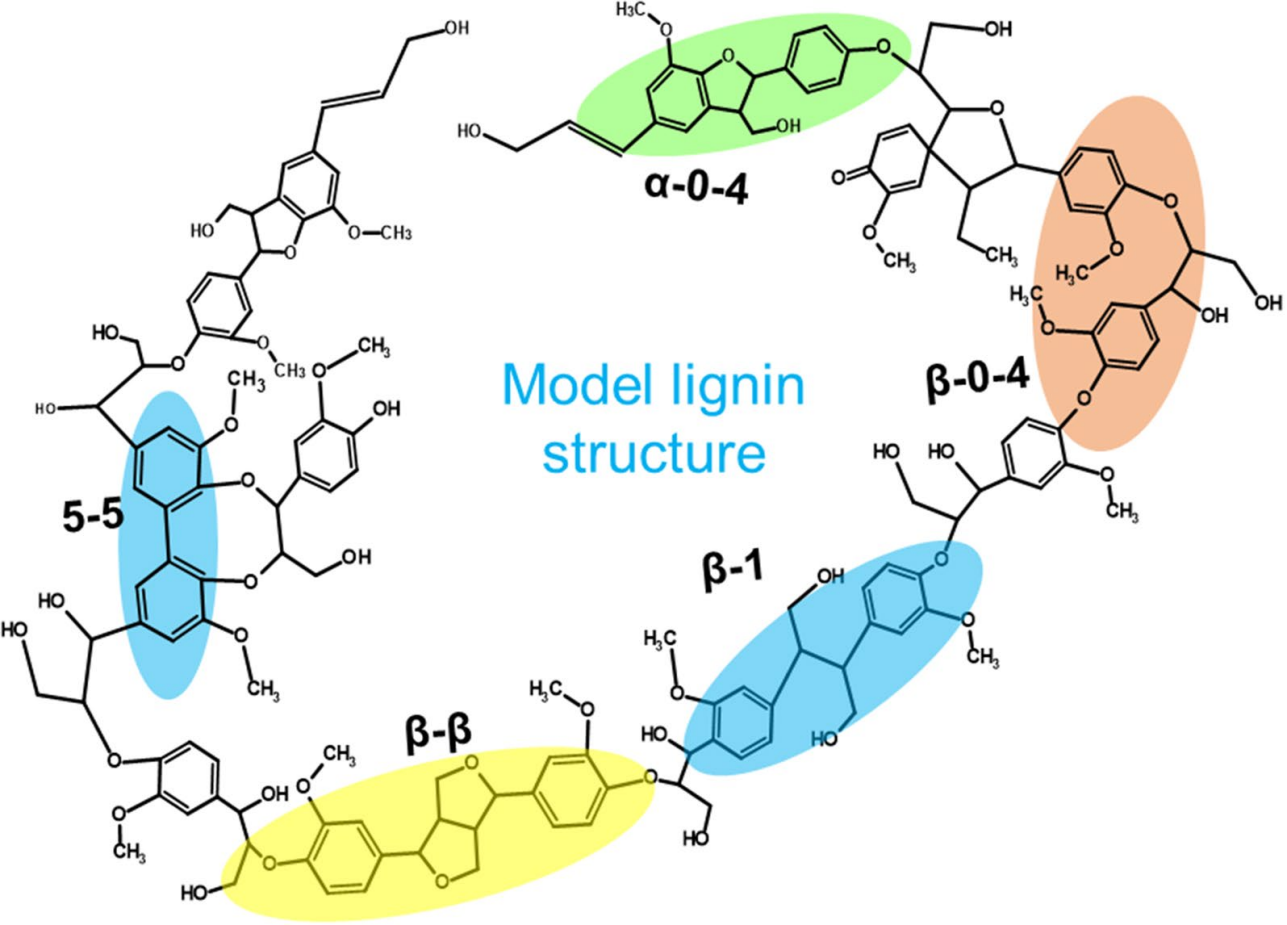
A model structure of lignin and common lignin linkages; adapted and modified from [125]

The plant biomass contains cellulose, hemicellulose, lignin, and a small number of extractives. Lignin is the most abundant natural aromatic compound. The functional groups of lignin include methoxyl, carbonyl, carboxyl, and hydroxy, linking to aromatic or aliphatic moieties in various amounts and proportions, which make lignin with different chemical structures [93, 94]. Up to 30% of the organic carbon on earth is sourced from lignin [95]. The typical lignin content of softwood is 24–33%, hardwood is 19–28%, and grasses is 15–25% [53, 96]. Various linkages in lignin molecules are shown in Fig. 4. The three-dimensional heterogeneous lignin structure is formed in plants by the radical polymerization of three aromatic precursors, such as p-coumaric, coniferyl, and sinapyl alcohols [97]. During the biosynthesis of lignin in plants, these monolignols are radically coupled with each other to form different inter-unit linkages, such as β-O-4 (45–50%), 5–5 (18–25%), β-5 (9–12%), β-1 (7–10%), α-O-4, (6–8%), and β–β (0–3%) [98, 99]. Due to its high content of phenolic precursors, lignin could potentially be a renewable source for aromatic chemical production [100, 101].

The most widely produced technical lignins are kraft, lignosulfonates, soda, and organosolv lignin. Some chemical properties of different lignins are presented in Table 1. Kraft lignin (KL) is produced by the sulfate pulping process, which accounts for nearly 85–90% of the world's total lignin production and is mostly burnt on-site for steam generation [102, 103]. In this process, the wood biomass is delignified by an aqueous solution of sodium hydroxide and sodium sulfide at 140–170 ℃ [51]. The recovered KL is not water soluble but highly soluble in an alkaline solution (Table 1). Moreover, KL has the highest number of phenolic hydroxyl groups due to the ample cleavage of *β-*aryl bonds. In addition, it has a significant amount of quinone, catechol, and carboxylic groups due to the delignification in the oxidative conditions [104]. The sulfite pulping process produces lignosulfonates (LSs), and the delignification is carried out at 120–180 ℃ in the presence of alkali metal sulfites and sulfur dioxide [105]. LS contains many anionic functional groups (Table 1), such as carboxylic, sulfonate, and phenolic hydroxylic groups [106–108]. The unique functional and structural properties of lignosulfonates make them excellent raw materials as dispersants [109], binders [110], adhesives [111], artificial HS [59], and cement additives [112, 113]. Due to a lack of economic viability, only 2% of lignin is utilized as a value-added product, such as vanillin. In contrast, the remainder is burned as a low-grade source of energy [114, 115]. Soda lignin (SL) is a by-product of pulping of mainly annual plants, like flax, straws, bagasse, etc. [116–118]. In this process, biomass is delignified by 13–16 wt.% of NaOH solution at 140–170 ℃ [51]. Soda lignin is highly pure due to its production in a sulfur-free pulping process. The applications of the soda lignins are suggested in phenolic resins, animal nutrition, and dispersants in polymer synthesis [102, 119–121]. Organosolv lignin (OL) is isolated from the black liquor of organosolv pulping, where biomass is digested at temperature ranges of 100 and 190 °C with organic solvents, such as acetic acid and formic acid ethanol [51, 122]. This lignin contains minimal sulfur content rendering it chemically pure [123, 124]. The potential applications of OL were suggested in ink formulations, varnishes, and paints [107] due to their lower molecular weight (Table 1). Also, OL gained attraction toward the preparation of wood adhesives and fillers [30].

### Structural similarities between lignin and HS

Recent studies support the similarities between lignin and HS. Chemically, both lignin and humic acid have similar functional groups, such as carboxyl, phenolic/aliphatic hydroxyl, and methoxyl, and, most importantly, aromatic moieties [55, 126, 127]. In soil's organic matter, polyphenols and aromatic carboxylic acids are believed to be formed from lignin degradation and several microbial syntheses [128]. Oxidized lignin-derived phenyl propane has also been confirmed to be present in the coal-based HS [129–131], suggesting that similar functional groups are shared between HS and lignins. Also, small aromatics identified by the pyrolysis of HS belong to lignin moieties [126, 127].

The oxidation (using CuO, KMnO_4,_ and H_2_O_2_ in an alkaline environment) products of humic and fulvic acids are similar to lignin aromatic moieties [132–134]. Yan et al. reported that 2–3 mmol/g of phenolic OH groups are found in different sources of HAs [134]. It was also suggested that the degradation products of HS are similar to lignin-based phenolic compounds [54, 135]. Other studies showed that structural units and some typical inter-unit linkages were preserved during the transformation of lignin into HS [136, 137]. A recent survey of composted grass lignin and humic acids showed that both materials have a similar range of phenolic OH contents (1.2–1.5 mmol/g) [138]. This study reported different carboxylic acid groups of ~ 0.8 and 2.3–2.7 mmol/g in lignin and HAs, respectively. Also, the methoxy groups of lignin were found to be almost 5 times as much as that of HA. These results support the earlier theories regarding higher carboxylic acid groups and demethylation in HS than in lignin. Interestingly, the alkaline nitrobenzene oxidation of the grass lignin and HA provided similar phenolic compounds, such as vanillin, vanillic acid, and syringyl and guaiacyl units, at varied amounts [138].

### Origin and challenges of HS

Humification is a complex biochemical process. It was observed that the polyphenol structures of the HS originate from the plant’s lignin [139]. The sources of some nitrogenous bonds may be due to the protein degradation of the microorganisms and the biomass from other dead animals. The characteristics of HS differ depending on the source and their extraction methods [129, 140]. Currently, the primary sources of HS are peat, leonardite, lignite, and river sediments, which are non-renewable sources [141]. Moreover, the excessive extraction of HS from natural sources may cause severe health hazards and ecological disturbance, including global warming, climate change, and land erosion in the long run, similar to coal mining [142]. It was reported that coal or lignite mining might release harmful organic substances that mix with surface water and drinking those water may cause severe kidney failure [142]. In addition, collecting HS from the river sediments would remove the under-water microorganisms, which can directly hinder the aquatic ecosystem. The helpful microorganisms facilitate the decomposition of dead biomass to adjust the ecological balance. Considering the drawbacks of natural HS resources, it is necessary to consider alternative ways for preparing HS from renewable sources, like lignin. As discussed, many HS are directly linked to biomass conversion (mostly lignins), and the artificial humification process can open windows of opportunities for utilizing lignin. However, the humification of technical lignins is yet to commercialize because of the complexity of the lignin structure. There are two primary methods for converting technical lignins to HS: direct oxidation [58, 59] and oxidative ammonolysis (OA) [60, 139].

### Humification of technical lignin by direct oxidation

Due to active hydroxyl groups, lignin acts as an excellent raw material for oxidative cracking and the production of various aromatic fine chemicals, including organic acids, aldehydes, and hydrophilic anionic lignin [37, 143–145]. The oxidation of lignin involves the depolymerization and fragmentation of the aryl ether bonds and other linkages [143]. Alkaline wet oxidation of lignin requires a high temperature (125–320 °C) and pressure (up to 2 MPa) in the presence of air or molecular oxygen [146]. Moreover, the post-treatment to separate the chemicals from the mixture is not economically feasible. According to the recent approaches, the direct oxidation of technical lignin toward transformation into HS-like materials can be categorized mainly in three ways, such as alkaline aerobic oxidation (AAO) of technical lignin, alkaline oxidative digestion (AOD) of lignocellulosic biomass by hydrogen peroxide, and Fenton reagent-based oxidation of lignin by hydrogen peroxide. Table 3 summarizes the different approaches of lignin and biomass oxidation toward artificial humification.

**Table 3:** Different oxidation approaches for lignin and biomass conversion for HS-like lignin material productions

Raw materials	Chemical/reagents used	Temperature (°C), time (min)	Carboxylic groups, mmol/g	Mw, g/mol	Application remarks	Refs.
LS	NaOH, H_2_O_2_/air	170–190, 180	NA	NA	Transformed LS to HA (77 wt.% yields)	[59]
LS	KOH, air/O_2_	NA	NA	NA	Increased corn root dry weight and chlorophyll by 18% and 45%, respectively at 1 mgC/L dose	[58]
KL	KOH, O_2_	195, 30	2.6	3500–4000	Increased fresh corn plant length, dry weight, and chlorophyll content by 27, 92 and 32%, respectively at 10 mgC/L dose	[144]
KL	FeSO_4_, H_2_O_2_	RT, 120	NA	NA	Increased seeds germinations and two folds of chlorophyll contents in leaves at 860 ppm dose	[131]
Giant reed	KOH/H_2_0_2_	50, ON	NA	NA	Enhanced tomato seed germination and early hypocotyl growth by 10% at 10 ppm	[157]
Giant reed and Miscanthus	KOH, H_2_0_2_	50, ON	1.02		Enhanced germination of maize seeds and root elongation increased by 50% at 10 ppm dose	[154]
Cardoon, Eucalyptus, and black poplar woods	NaOH, H_2_0_2_	50, ON	0.4–1.4	NA	Increased maize seedling growth by 72% at 10 ppm dose	[158]

*NA* not available, *RT* room temperature, *ON* overnight

### Alkaline aerobic oxidation (AAO) of lignin

**Fig. 5:**
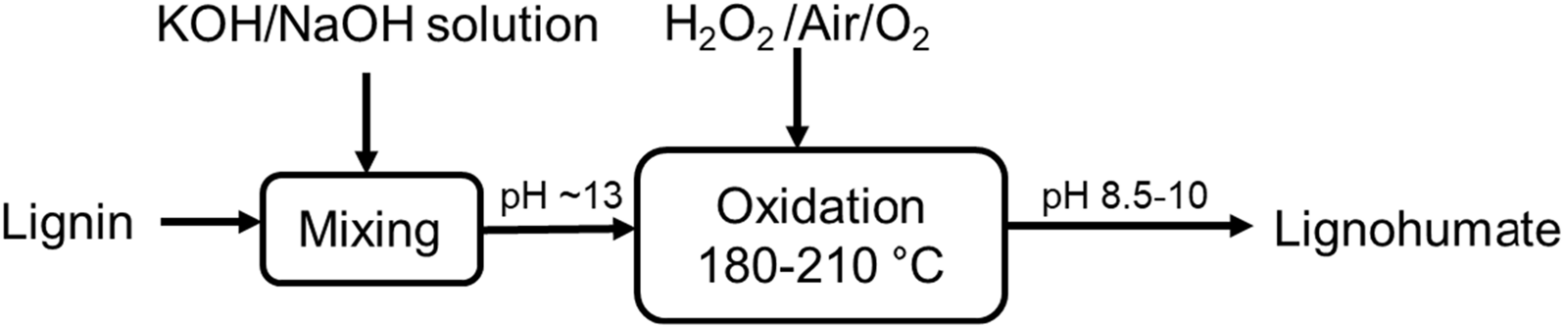
A schematic flow diagram of alkaline aerobic oxidation for lignohumate production from lignin

Figure 5 demonstrates the schematic flow diagram for producing artificial lignohumate (ALH) from technical lignin by AAO [59, 144]. In this process, lignin is dissolved in alkaline solutions, such as KOH or NaOH (as a catalyst), to activate the phenolic OH groups of lignin and later oxidize by air/oxygen or hydrogen peroxide. After the reaction, the product can be used directly, either in liquid or in solid form. However, the AAO generated by NaOH treatment may need to be purified by dialysis as Na^+^ may increase salinity and inhibit plant growth when applied as a fertilizer [144].

**Fig. 6:**
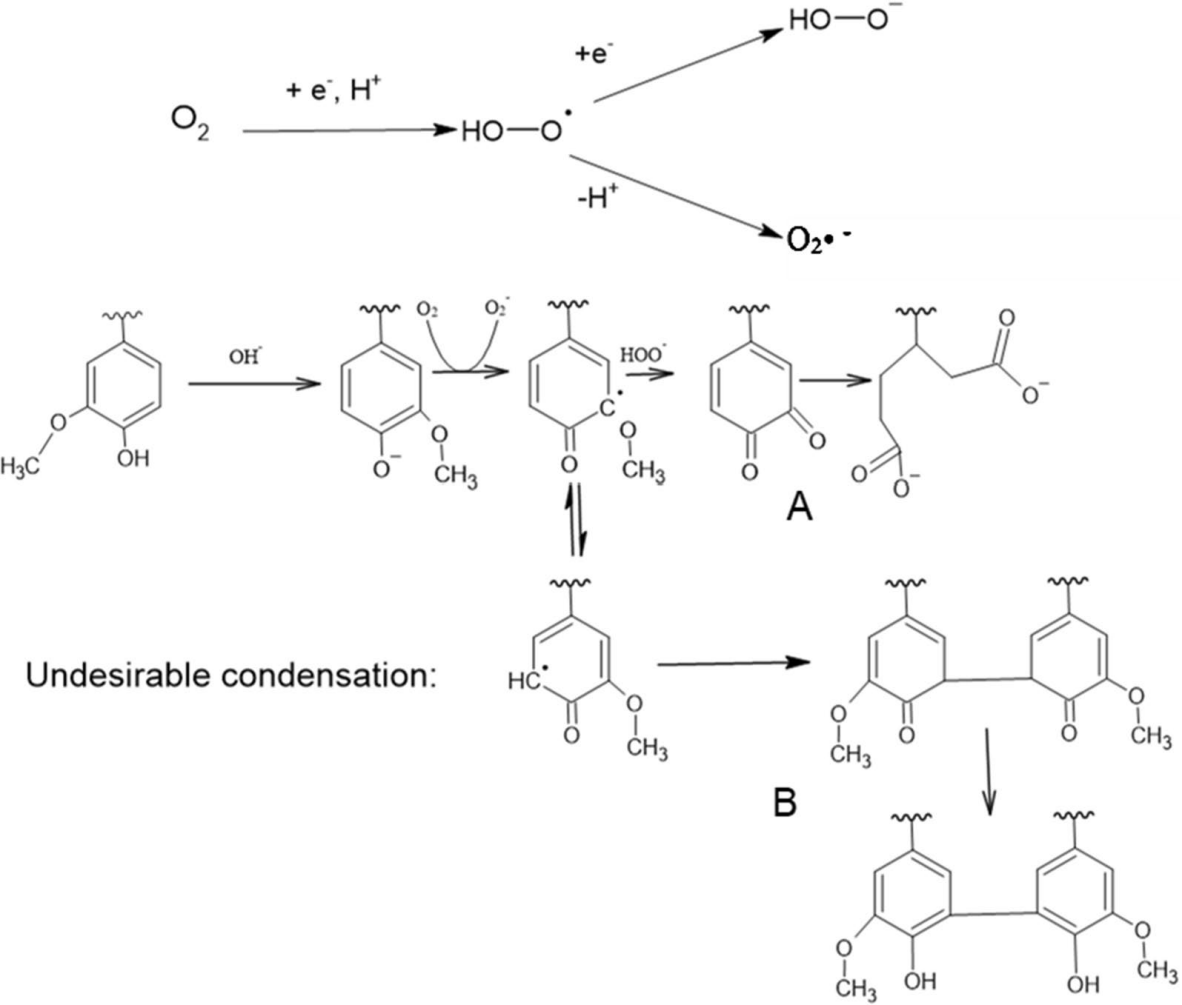
Reaction pathways for the alkaline aerobic oxidation of lignin adapted from [144, 148, 155, 156]. Route A: degradation (simplification). Route B: undesired coupling)

Figure 6 represents the simplified mechanism of AAO of lignin toward forming HA-like materials. Initially, lignin’s free phenolic hydroxyl groups are ionized to produce phenolates in an alkaline environment. Then, O^2−^ reacts with phenolate and forms phenoxyl radicals, i.e., the first oxidation product. The superoxide radical anion (O^2^·−) attacks in the meta position and breaks the methoxy groups of lignin to convert into quinones [144, 147]. Further oxidation leads to aromatic ring cleavage and the formation of dicarboxylic acid (or any orthoquinone compounds) [147, 148]. Route B in Fig. 6 represents the undesirable coupling of phenolate ions to form biphenyl compounds, which leads to the repolymerization of lignin.

Naturally, the HS are enriched with organic acid groups. Therefore, the fundamental target of alkaline aerobic oxidation is to convert the phenolic and aliphatic hydroxyl groups of lignin into carboxylic groups [50]. Significant structural changes are observed during this oxidation, such as decreasing methoxyl, aliphatic, and phenolic hydroxyl groups, while increasing aliphatic and aromatic acid groups [144]. These anionic groups increase the hydrophilicity of the oxidized lignin materials and play a significant role in mineral transportation toward the roots [144]. In one study, lignosulfonate was oxidized with hydrogen peroxide in an aerobic system, which increased the mass shares of HA up to 77% [59]. It was reported that similar to naturally occurring HS, the oxidized KL and LS showed positive physiological effects on plant growth, such as increased length, dry weights, carbohydrate/sugar synthesis in the plants, and chlorophyll contents on the leaves [58, 144]. However, the AAO of lignin generates a wide range of phenolic monomers and derivatives, which not only improve the aforementioned physiological effects but also stimulate hormonal activities, such as auxin (IAA) and Gibberellin (GA) [58, 149–151]. However, depending on the structural conformation and concentrations, some phenolic acids may show inhibitory effects on plant growth and other bioactivities [152–154].

### Alkaline oxidative digestion (AOD) of biomass

**Fig. 7:**
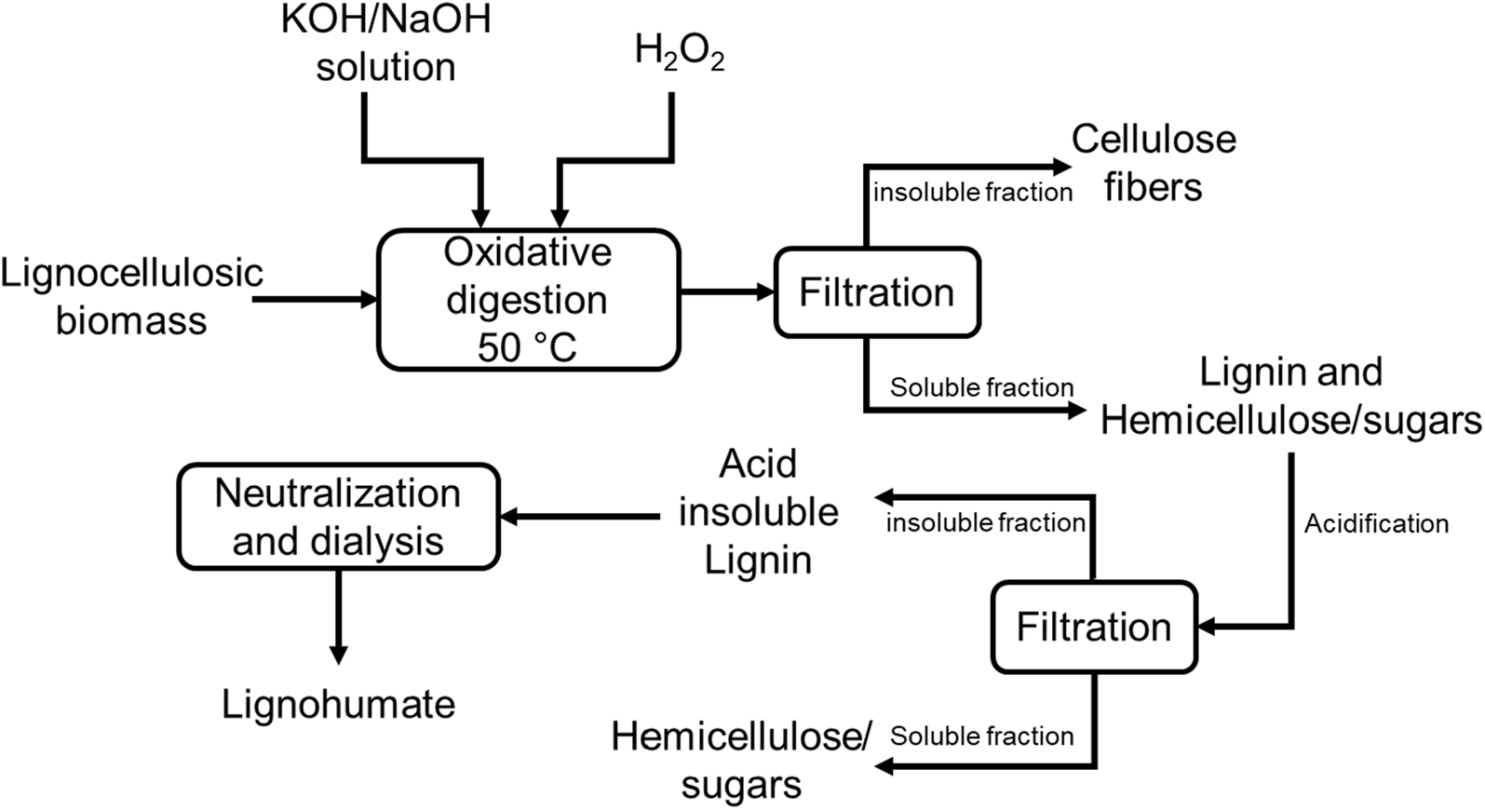
A schematic flow diagram of alkaline oxidative digestion for lignohumate production from lignin

In another pathway, lignocellulosic biomass was modified to water-soluble lignin via an alkaline oxidation digestion procedure for producing HS-like materials [157–159]. A schematic flow diagram of this process is presented in Fig. 7. In this system, biomass is allowed to digest in an alkaline (KOH/NaOH) oxidative environment in the presence of an oxidant, e.g., hydrogen peroxide. After the digestion, the insoluble cellulosic fibers are removed by filtration, and the filtrate is acidified to separate hemicelluloses/sugars from lignin. After that, the separated lignin is suspended in water and neutralized to get water-soluble fractions, which are considered lignohumate. The oxidative reaction mechanism on lignin should follow a similar path as alkaline aerobic oxidation.

Transforming biomass through the AOD process has a few advantages, such as direct use of biomass for conversion, low operating temperature (50 ℃, overnight), and obtaining cellulose fibers as by-products. Moreover, monomeric toxic phenolic compounds (also known as phytotoxic chemicals) can be achieved due to the acidification step (Fig. 7). The carboxylic acid groups can be achieved up to 1.4 mmol/g [154, 157, 158]. The bioactivity of the extracted lignin toward any plant depends on the hydrophilicity of lignin samples. Several studies on the oxidative digestion of biomass showed that non-wood water-soluble lignin (i.e., isolated from giant reed, miscanthus, cardoon, etc.) had higher hydrophilicity and bio-stimulating performance on plant growth [154, 157, 158]. On the other hand, oxidized eucalyptus lignin was the least effective for plant stimulations following this oxidation method, which may be attributed to their poor hydrophilicity due to less hydroxylated long aliphatic chain, inhibiting the release of bioactive molecules to the aqueous environment [158].

### Fenton reagent-based oxidation of lignin

**Fig. 8:**
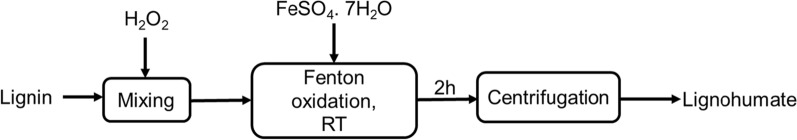
A schematic flow diagram of Fenton reagent-based oxidation for lignohumate production form lignin; *RT* room temperature

A new method was developed for the oxidation of lignin, e.g., kraft lignin, by hydrogen peroxide in the presence of a Fenton reagent catalyst at room temperature [131]. Figure 8 represents the schematic flow diagram of this method. In this process, lignin is mixed with a hydrogen peroxide solution. After the mixing, the solution is oxidized at room temperature in the presence of iron (ii) sulfate heptahydrate. After the oxidation reaction, the solution is centrifuged and washed several times with deionized water to remove any unreacted chemicals and some toxic phenolic compounds. The solid residue (oxidized lignin) is lyophilized for further application as lignohumate.

The Fenton reagent-based lignin depolymerization is considered a nonspecific oxidation process. The Fenton reactions would allow lignin particles to mimic commercial HA because of the presence of the oxidized iron-based inorganics deposited on the lignin-based products. The primary goal of this oxidation is to increase the O/C ratio, which would indicate the formation of oxygenated functional groups, such as quinones, carbonyl, and carboxylic acid groups in the oxidized lignin. The outcomes of this process mainly depend on the organic structures of lignin and the ratio of hydrogen peroxide and iron (ii) sulfate [131, 160]. However, the Fenton-induced oxidation may generate some phytotoxic phenolic compounds [161]. Therefore, a post-separation of the soluble fractions (containing phenolics) is recommended to obtain a purified product.

### Humification of lignin by oxidative ammonolysis (OA)

The artificial humification can be carried out by the OA process of lignin, which can incorporate a considerable amount of nitrogen in the humified lignin in different forms. Generally, soil’s organic matter, such as HS, must have nitrogen for efficient biodegradation affinity. Research showed that a C/N ratio under 20 facilitates biological degradation [60], whereas a higher value than 25 can hinder the degradation process. Natural humification could be conducted artificially by reacting technical lignin with ammonium hydroxide/ammonia solution, increasing the C/N ratio and crop productivity [139].

**Fig. 9:**
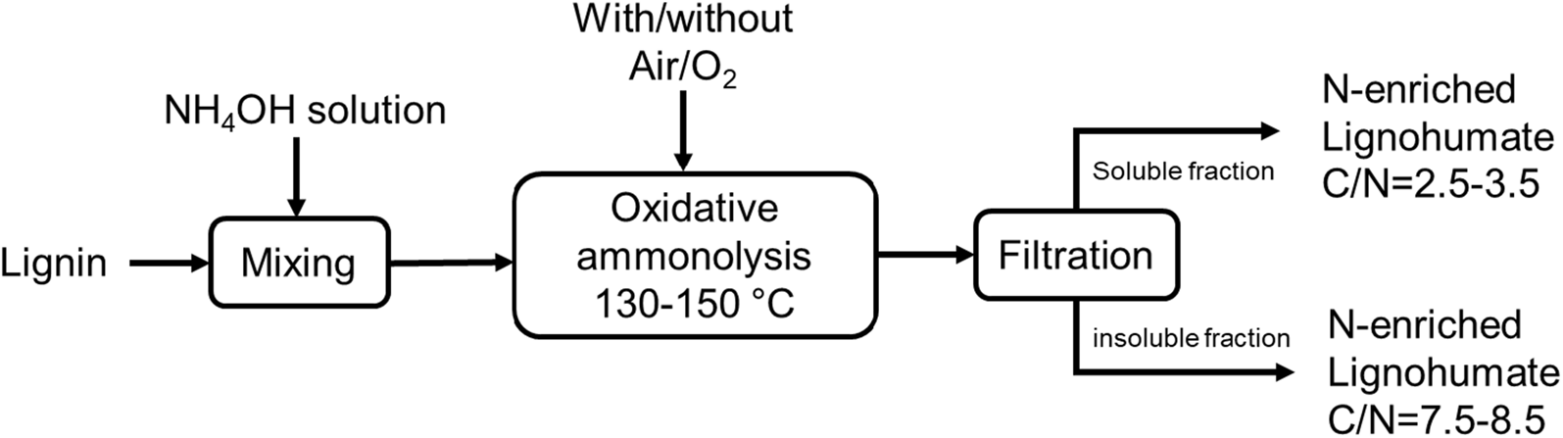
A schematic flow diagram of oxidative ammonolysis for N-enriched lignohumate production from lignin

**Fig. 10:**
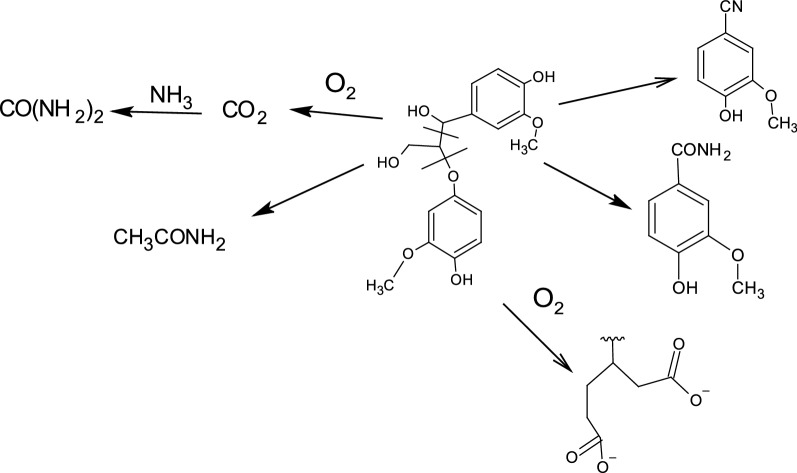
Model reaction scheme for the oxidative ammonolysis of lignin; adapted and redrawn from [139]

Figure 9 demonstrates the preparation of nitrogen-enriched lignohumates (N-ALHs) following the oxidative ammonolysis (OA) process [56, 57, 162]. In this method, lignin is suspended in different concentrations of NH_4_OH solution. The reaction is carried out in the temperature ranges of 130–150 ℃ and treated with or without any oxidants (air/oxygen). The water-soluble and insoluble parts are separated after the reaction and can be utilized as different grades of fertilizers [60]. The reaction mechanism of the OA system is shown in Fig. 10. It is seen that lignin fragmentation occurs at the aliphatic side chain during the OA process resulting in the cleavage of β-O-4 linkages [139]. The aromatic part of the lignin provides substituted acid derivatives, such as amide and nitrile compounds. Due to the oxidizing environment, some aromatic rings of lignin are degraded to convert into aliphatic dicarboxylic acids through quinone formations. Later, these aliphatic acids may react with available ammonium ions to form their salts. In addition, as the OA is carried out at a fairly high temperature and pressure, the produced CO_2_ can react with the unreacted ammonia gas to produce urea as the final product [139].

Different approaches to lignin modification by OA are listed in Table 4. The primary target of the OA process is to incorporate nitrogen into lignin molecules in the form of ammonium salts, amides, and urea-type structures, but not amines or heterocyclic [56, 163]. The transformation of KL, LS, and OL into N-enriched fertilizers via the OA was exploited in the past [56, 57, 60, 61]. KL showed a higher reactivity toward OA among all lignin due to abundant phenolic hydroxyl groups [60]. Some studies investigated the effect of the reaction parameters on nitrogen incorporation in lignin [56, 57, 61]. It was observed that lignin's methoxyl and carbon content would decrease with nitrogen incorporation during the OA reaction [57, 60]. Interestingly, an increase in the reaction solution's pH increased the lignin oxidation rate and consequently increased its nitrogen incorporation [61]. It was stated that when increasing the concentration of ammonium hydroxide from 0.4 to 1.6 M, the lignin solubility in the reaction mixture increased to almost 75%, thus enhancing the reactivity toward OA and increasing the nitrogen incorporation [56, 61]. Also, the rate determining step of the OA reaction is the oxidative cleavage of the non-phenolic moieties and the oxidation of aromatic rings because the rate of nitrogen incorporation is directly related to these steps and directly proportional to oxygen pressure [56].

**Table 4:** Different approaches for the modification of lignins by OA toward N-ALH

Raw materials	Chemicals/reagents	Temperature (℃), time (min)	C/N	Application remarks	Refs.
KL, LS, OL	NH_4_OH, O_2_	150, 120	4–7	Increased Sorghum productivity by 3 times	[60]
OL	NH_4_OH, O_2_	130, 15–1455	3–5	Increased 63% nitrogen incorporation	[56, 57]
OL	NH_4_OH, O_2_	100, 15–180	NA	Increased 67% nitrogen incorporation at pH 11	[61]

The fertilizing effects of these N-ALH were also studied earlier. In one study, up to 14% nitrogen was incorporated into the lignin and used as fertilizer in the pot experiments [60]. The earlier studies on the OA process showed that the C/N ratio could be decreased to 3–7 (Tables 1 and 4). Meier et al. studied the effects of N-lignin (modified by OA, C/N 4–7) on Sorghum plants at the dose rate of 1385 kg/ha (180 kg/ha of nitrogen content), and the results showed a crop yield increase of 82%. Another study showed that applying artificial lignohumates (N-enriched, total N content 10–24%) on different woody plants increased the green mass of the plants by more than 50% and decreased the nitrogen leaching by nearly 75% compared to commercial urea [139]. Therefore, transforming technical lignin into nitrogen fertilizer through the OA could be a promising route in the agricultural field due to the available organic carbon and nitrogen. In addition, the N-lignin's oxygenated part (i.e., carboxylic ends) would participate in mineral transportation.

### Potential applications of humified lignin

#### Soil treatment

Although natural HS are used mainly as soil conditioners, there are other potential applications for HS in soil. HS help segregate the compactness of soil structures, reduce water evaporation from the soil surface, and have a role in transporting micronutrients from ground to plants [164]. Artificial humified lignin derivatives may have these unique properties too. The ALH with similar physicochemical properties will be an excellent alternative as a soil stimulator [58, 144, 157, 158, 164]. As controlled alkaline oxidation of lignin results in increased aromatic/aliphatic OH and carboxylic OH contents (Table 3) in the products, they should function similarly to natural HS [59, 144]. In this context, ALH may have potential applications for soil loosening, decreasing the bound water evaporation rate, and transporting essential nutrients to plants.

**Fig. 11:**
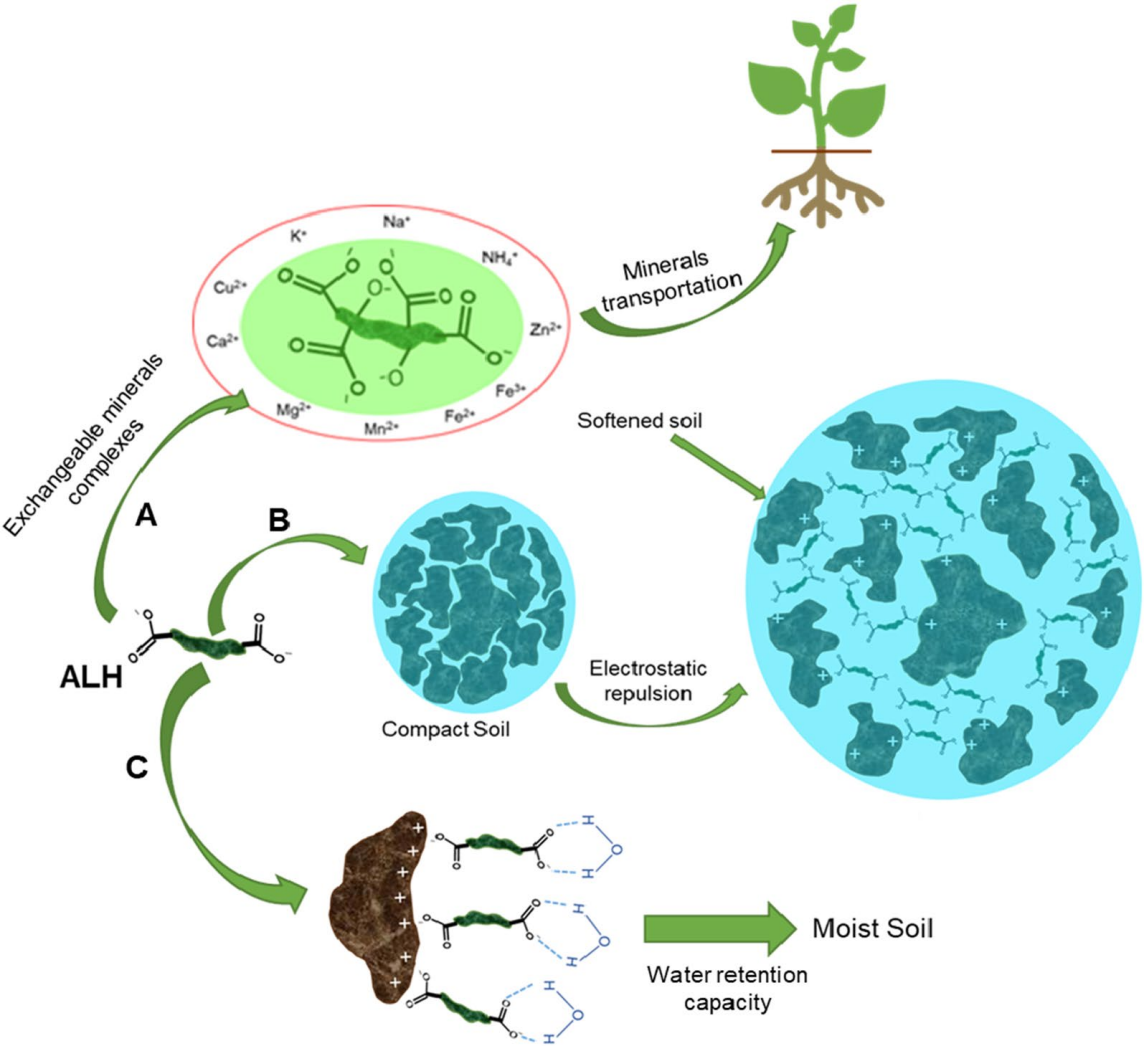
A schematic representation of mineral transportation, soil conditioning, and water retention capabilities of ALH; adapted and modified from [165, 175]

Figure 11 demonstrates a model mechanism of ALH in soil. Route A describes that the carboxylic and hydroxyl groups of ALH will dissociate into their ions, and the hydrophilic ends will exhibit the chelating behavior. The anionic hydrophilic ends will form unstable complexes with the essential minerals available in the soil, such as Na^+^, K^+^, Ca^2+^, M^2+^, Fe^2+^, and Fe^3+^, by electrostatic attraction [165]. It was reported that the mineral transportation by natural HS would occur differently by low molecular weight (LMW, < 3500 g/mol) and high molecular weight (HMW, > 3500 g/mol) fractions [166]. The HMW fractions (HA) of HS stimulate the root plasma membrane and enzyme activity and increase plant growth, while LMW fractions (FA) are directly co-transferred into the plant's roots [166–168]. In addition, the LMW fractions were greatly responsible for NO_3_^−^ uptake and nitrogen metabolism [169, 170]. The LMW fractions of HS have better mineral binding capacity than HMW, improving nutrient absorption by roots due to the relative abundances of oxygenated functional groups (carboxylic and phenolic OH groups) [166, 171, 172]. Nardi and co-authors reported that the LMW fractions stimulate hormonal activity (i.e., auxin, gibberellin, and cytokinin). However, the HMW fractions controlled the availability and activity of LMW on plant metabolism [169]. Therefore, the ALH that is obtained from AAO can be fractionated as LMW fractions and HMW fractions for specific applications.

Route B demonstrates the dispersion ability of ALH on the soil. Generally, ideal soil contains 45, 5, 25% of minerals, organic matter, and air, respectively; and the rest is water [173]. If the soil minerals increase to 69%, it will decrease the organic matter and air to 1 and 5%, respectively, resulting in a compact soil structure [173]. As a result, water penetration into the soil would be hampered. Therefore, the dissociated minerals (positive and negative mineral ions) would attract each other to form salts. In this case, when ALH is used, the organic content would be increased, which would help interact with the positive mineral ions and possibly adsorb them due to the presence of strong anionic hydrophilic groups. In this way, ALH would restore the negative ions into the soil. Moreover, the ALH would create electrostatic repulsion due to the anionic hydrophilic ends, and phenolic ends would enhance the steric hindrance to disperse the soil particles resulting in untied soil [174]. The ALH derived from AAO should have more negative charge density due to having higher carboxylic acid groups (Table 3) than ALH from AOD. Therefore, AAO-derived ALH should exhibit more increased dispersibility in soil.

Route C represents the water retention capacity of ALH. Due to the hydrophilic anionic functional groups (i.e., carboxylic groups) (Table 3), the ALH would be adsorbed by the positively charged minerals in the soil, and the other ends would hold the water molecules because of the electrical attraction [175, 176]. Therefore, the ALH derived from AAO and AOD would be suitable for increasing the soil's water retention capacity.

On the other hand, the N-ALH derived from the OA process may be appropriate as a fertilizer since it contains a lower C/N ratio (Table 3). The direct application of N-ALH has been studied for crop productivity and slow-release fertilizing ability [60, 139]. However, other effects on soil, such as mineral transportation, soil texture, and water retention capacity, were not yet studied for the N-ALH.

### Medicinal application

Due to their antiviral [177, 178], anticarcinogenic [179], antibacterial, antioxidant, anti-inflammatory, and antiseptic properties [164, 180], the medicinal usage of HS has been practiced for centuries [164]. The antioxidant properties of lignin-derived materials have also been reported due to the availability of phenolic and acidic (aliphatic and aromatic) groups, which have chelating and radical scavenging properties [32, 181]. On the other hand, low molecular weight (i.e., 1500 g/mol) fractions of HS show inhibiting effects against HIV-1 in vitro [164]. The anticarcinogenic properties of FA fractions were also reported earlier [182]. In addition, an earlier study reported that the oral consumption of HA by domestic animals could reduce the cholesterol, lipids, and glucose content and increase the red blood cells and hemoglobin in the animal bodies [183]. One recent study also reported the potential antiviral effects of natural HS against the recent COVID-19 virus [184].

In this context, the smaller molecular weight fractions of the ALH generated from the direct alkaline oxidation (both AAO and AOD) of lignin products (i.e., primarily oligomeric phenolic derivatives) can be utilized for medicinal applications. As stated above, the ALH is capable of complexation with metals, such as iron, due to the abundant of phenolic and carboxylic acid groups [131]. Similar to FA, ALH can be a novel compound to improve the rate of iron adsorption in blood and increase the number of red blood cells [164]. Antioxidant medications reduce the risk of several diseases caused by oxidative stress, typically brought on by free radicals like reactive oxygen species (ROS), such as superoxide anion, hydroxyl free radical, and hydrogen peroxide [185]. ALH can be a potential substance as an antioxidant by neutralizing these ROS due to their heterogeneous aromatic compositions (i.e., phenolics and quinones) and supramolecular structure [32, 181, 185]. The reactive phenolic moieties of oxidized lignin might cause bacterial and microbial cell death [185].

Moreover, the acidic functional groups (aliphatic or aromatic) of the ALH would reduce the cell binding of different viruses (i.e., HIV) [186]. Although the chemical properties between natural HA and AAO/AOD-derived ALH are comparable, extensive studies are needed to examine the medicinal effects of the ALH materials. Finally, for medical applications, the post-purification of the ALH is highly recommended for removing the excess alkali and other toxic chemicals (i.e., phenol) generated from the reactions [187].

### Wastewater treatment

Wastewater treatment by HA has been studied extensively [188–192]. Similar to its action in soil, it can develop complexes with heavy metal ions in solution systems, reducing the toxicity of drinking water, industrial wastewater, and surface water. The mechanisms of HS for wastewater treatment depend on factors, such as the nature of the HS (particularly the fulvic and humic acid content), soil chemistry, and water's chemical properties, such as acidic or alkaline. Like HS, ALH can be an alternative product to remove these heavy metals and other suspended particles, such as oil, grease, and certain organic compounds from water. The long lipophilic aliphatic chain and hydrophilic ends should have excellent surfactant properties that help remove oil and greases [193, 194]. The anionic characteristics of the carboxylic acid groups on ALH should demonstrate their high cationic exchange capacity, enhancing the formation of insoluble complexes with the polyvalent metal cations. The complexation of heavy metals, such as lead (Pb), copper (Cu), cadmium (Cd), nickel (Ni), cobalt (Co) zinc (Zn), iron (Fe), and aluminum (Al), with the ALH is possible if the ALH has a desired carboxylic content. The metal complexation is highly pH (pH 4–8) dependent and forms strong chelates with the metal ions having oxidation states of + 2 [195]. In addition, a high molecular weight (14,000–33,700 g/mol) ALH would be more effective for wastewater treatment [196, 197]. Although the current approaches (i.e., aerobic oxidations) of transforming lignin to ALH attain sufficient anionic functional groups, the molecular weights are significantly reduced (Table 3), making them less effective for heavy metal removal applications. However, extensive research on new method development is necessary for the scope of wastewater treatment by ALH.

### Challenges and future directions of lignin modification toward humification

Generally, the main drawback of lignin valorizations is claimed to be its complex heterogeneous aromatic structures, while it is a blessing in terms of its transformation toward humification. In the direct oxidative process of lignin, a high temperature (170–195 ℃) is required to break down the lignin skeleton and reduce the molecular weight of lignin significantly, which may limit the application of the produced materials. This is because the high molecular weight fraction of HS is known to have higher performance for heavy metal removal and soil softening (dispersibility) [198]. Therefore, a milder reaction condition maintaining the lignin structure more intact would be preferred to help protect the linkages and oxidize the lignin structure selectively.

It was reported that the oxidation of lignin would produce phenolic monomers, such as protocatechuic acid, hydroxybenzoic acid, and p-coumaric acid. Those phenolic compounds are known as potential allelopathic agents (phytotoxic chemicals) and inhibit plant growth [58, 153, 199–202]. The negative effects of those phenolic compounds depend on their used concentrations and their chemical structure and specific plant species [152, 203]. The direct oxidation methods of lignin for humification may require a separation process to remove the phytotoxic compounds (Figs. 7 and 8), which may be costly. Therefore, introducing new selective oxidizing catalysts or technological advances in the oxidation process may be required to reduce the production cost of such chemicals in converting lignin to HS.

Naturally occurring HS are enriched in carbon and nitrogen [139, 204]. Few studies claim that natural sources of HS, such as lignite, i.e., one of the major coal sources for commercial HA, contain significant amounts of iron in polyphenol − Fe complexes [205–208]. A past study revealed that HS and lignin-derived HS have similar levels of carbon [144]. However, none of the other plant essential nutrients (K, Fe, Ca, N, P, etc.) are present in ALH, which is one of the main limitations of using artificial HS as organic fertilizers and soil stimulators. Incorporating inorganic minerals into ALH is another critical stage to transforming lignin into HS-like materials. Natural HS are found in complexes with different transitional metals, like Fe [209]. Learning from this, Fenton-based single-staged oxidation under mild conditions can be an example of converting lignin into artificial HS with Fe complexes. In this context, Jeong et al. reported that a Fenton-based one-pot advanced oxidation was employed to mimic fungus-driven lignin humification and incorporate iron into the oxidized lignin samples [131]. In addition, Fenton reagent-based alkaline (KOH) oxidation can enhance the lignin reactivity and conversion to the HS-derived product.

Currently, there are some challenges with lignin reactivity in the OA processes. Meier et al. reported that lignosulfonates and kraft lignin showed higher reactivity than any other lignins for OA [60]. In contrast, the ASAM (Alkaline Sulfite Anthraquinone and Methanol) lignin was not suitable for this process due to its high degree of sulfonation, low molecular weight, and high ash content. Moreover, current approaches of OA were carried out with NH_4_OH and an oxidant, such as air/oxygen [60]. Due to their available nitrogen, OA-modified lignins are generally limited to fertilizing applications. In addition to NH_4_OH, KOH, and other alkalis can be used to improve the lignin dissolution and enhance lignin's oxidation reaction [59]. This way, the modified lignin would be enriched with nitrogen in different forms. KOH would facilitate the formation of carboxylic acid groups [144], which could make new routes for producing HS-like lignin. Moreover, the global HA market is expanding day by day, mainly in the agricultural sector. It was reported that the market value of HA in the agricultural field was around USD 365 million, and it is projected to reach up to USD 934 million by 2030 [210]. Currently, HA production mainly depends on natural sources (i.e., coal, peat, lignite river sediments, etc.), which is neither a sustainable process nor environment friendly. Therefore, the chemical transformation of lignin materials toward artificial humification can be a potential route considering the current HA's renewability, sustainability, and environmental concerns.

## Conclusion

Naturally produced HS contain insoluble humin, alkali-soluble HA, and water-soluble FA fractions. HA has been widely used as a soil conditioner due to its wide range of oxygenated functional groups, such as phenolic hydroxyl, quinones, and carboxylic acid. Past research showed that those functional groups might have originated from lignin decomposition in natural HA. As several physicochemical properties, such as solubility, phenolic hydroxyl, and carboxylic acid groups, of lignin and HA are similar, the chemical transformation of lignin to HS is possible. The most popular method to transform lignin/lignocellulosic biomass into HS is alkaline oxidation (AAO and AOD). These processes' primary goal is to increase the lignin materials' hydrophilicity by converting aliphatic/phenolic hydroxyl groups to carboxylic acid groups. On the other hand, OA aims to incorporate nitrogen into the main lignin structure in different forms, such as ammonium ion, amide, and nitrile. Although the AAO can be readily applied for lignin conversion, the costs associated with the post-purification of the product for eliminating phytotoxic chemicals generated during the oxidation process are challenging. Finally, to meet the demand for generating high-quality lignin-derived HS for applications in soil, wastewater treatment, and medicine, more research is needed to mitigate the challenges of incorporating other inorganic mineral nutrients (i.e., K, Fe, N, etc.) into lignin-based HS. Also, the post-purification of lignin-derived HS is required for eliminating toxic chemicals while maintaining desired characteristics, such as molecular weight and carboxylic acid groups.

## Data Availability

Data of this work will be available upon request from the corresponding author.

## Abbreviations

HS: Humic substances
HA: Humic acid
FA: Fulvic acid
KL: Kraft lignin
LS: Lignosulfonate
SL: Soda lignin
OL: Organosolv lignin
AAO: Alkaline aerobic oxidation
AOD: Alkaline oxidative digestion
ALH: Artificial lignohumate
HT: Hydrothermal
FR: Furfural
HMF: 5-Hydroxymethyl-furfural-1-aldehyde
IAA: Auxin
GA: Gibberellin
OA: Oxidative ammonolysis
N-ALH: Nitrogen-enriched ALH
LMW: Low molecular weight
HMW: High molecular weight
ROS: Reactive oxygen species
ASAM: Alkaline sulfite anthraquinone and methanol
